# Important Announcement

**Published:** 1898-06

**Authors:** 


IMPORTANT ANNOUNCEMENT.—*To the Medical Profession:* In order to meet the competition of the remaining three Texas medical journals, the subscription price of which is $1 per annum, and to still hold the lead, we have decided to put the subscription price of the Red Back on a level with its competitors.

We beg to announce, therefore, that, beginning with next number, July, 1898, the first number of volume XIV, the subscription price will be *one dollar per annum*, payable in advance. Those who have already renewed their subscription for volume XIV, remitting $2, will be credited with two years' subscription.

This is certainly "a great deal of sugar for a cent," as the North Carolina "Reb." said when the girl at the depot kissed him for his mother. It is a sacrifice. We never yet heard any one say that the Red Back is not worth $2 a year; but we have had many assurances by letter that some one article in some one issue was worth five times the price of a year's subscription.

We say "the remaining three medical journals in Texas." The Red Back has in the thirteen years of its successful experience—during which time it has had a most generous support, paying a good return on the labor expended upon it—witnessed the rise, progress (?) and decline (and "fall off") of quite a number of medical journals, but still, enough remain on the surface to stimulate the Red Back to renewed industry; and you may just bet your bottom dollar that it is going to hold the lead if we have to give a chainless bicycle or a U. S. A. commission as colonel with each $1 subscription!

Send along your one dollars for renewal of subscription from July, '98, to June 30, '99, volume 14, or $2 to June 30, 1900.

The following is in order, reproduced for the information of those who have not had the honor of making the acquaintance of the "joy" or the "terror" (as the case may be).

"A Joy to its Friends and a Terror to its Foes.—A journal that gladdens the heart and delights the understanding every month is the *Texas Medical Journal*, otherwise known as the "Red Back." As Chimmie Fadden would say, *it is up to de limit.* Standing for the ethical in both medicine and journalism, it is the uncompromising foe to the medical pretender and adventurer. It it a joy to its friends and a terror to its enemies; *suaviter in modo, fortiter in re.* There is but one *Texas Medical Journal*, and Bro. Daniel (Austin, Texas), is the editor thereof. May its vigor never perish, nor its complexion fade.—*Atlanta Medical and Surgical Journal, January, 1897.*

